# Patterns of extra-territorial nest-box visits in a songbird suggest a role in extrapair mating

**DOI:** 10.1093/beheco/arac111

**Published:** 2022-12-23

**Authors:** Peter Santema, Bart Kempenaers

**Affiliations:** Department of Behavioural Ecology and Evolutionary Genetics, Max Planck Institute for Ornithology, Seewiesen, Germany; Edward Grey Institute, Department of Biology, University of Oxford, Oxford, UK; Department of Behavioural Ecology and Evolutionary Genetics, Max Planck Institute for Ornithology, Seewiesen, Germany

**Keywords:** blue tit, *Cyanistes caeruleus*, dispersal, extrapair paternity, prospecting

## Abstract

Many animals make visits outside of their territory during the breeding period, but these are typically infrequent and difficult to observe. As a consequence, comprehensive data on extra-territorial movements at the population-level are scarce and the function of this behavior remains poorly understood. Using an automated nest-box visit tracking system in a wild blue tit population over six breeding seasons, we recorded all extra-territorial nest-box visits (*n* = 22 137) related to 1195 individual breeding attempts (761 unique individuals). Sixty-two percent of breeders made at least one extra-territorial visit between the onset of nest building and the day of fledging of their offspring, and individuals visited another nest-box on average on 11% of the days during this period. Visit behavior differed clearly between the sexes, with males making over three times as many extra-territorial forays as females. There was a strong overall seasonal decline in visit behavior, but this was sex dependent, with females showing a strong reduction in the number of extra-territorial visits before the onset of egg laying and males showing a strong and sudden reduction on the day their offspring hatched. The likelihood of visiting a particular nest-box declined sharply with the distance to that box, and blue tits almost exclusively visited direct neighbors. Individuals were more likely to have extrapair offspring with an individual whose box they visited, but they were not more likely to disperse to a box they had visited. Thus, our results are inconsistent with the hypothesis that extra-territorial nest-box visits serve to inform dispersal decisions, but suggest that such visits are linked to extrapair mating opportunities.

## Teaser

In blue tits, visits to nest-boxes outside a bird’s territory vary in time and space, and suggest a potential role in extra-pair mating. We used an automated monitoring system to record all extra-territorial nest-box visits by birds over six breeding seasons. Visits were primarily performed by males, mainly took place during the early breeding period, and were restricted to close neighbors. Extra-territorial box visits predicted patterns of extra-pair paternity, but not patterns of breeding dispersal.

## INTRODUCTION

Many animals live in territories that they defend against conspecific intruders for at least part of their annual cycle (Maher and Lott 2000). In many of these species, individuals are occasionally observed leaving their own territory to visit the territory or nest of a conspecific. Such extra-territorial visits are common in birds (e.g. Reed et al. 1999; Schlicht et al. 2015) and mammals (Doolan and Macdonald 1996; Kamler et al. 2019), but have also been reported in reptiles (Aragon et al. 2006), amphibians (Gautier et al. 2006), and insects (Seeley and Buhrman 2001; Francks et al. 2007; Canonge et al. 2011). Extra-territorial visits have been related to dispersal behavior, territory acquisition, age at first breeding, and reproductive success (Zack and Stutchbury 1992; Badyaev et al. 1996; Schjørring et al. 1999; Cam et al. 2002) and can thus have important fitness benefits. Extra-territorial visits are likely also costly to perform, as they are associated with energetic costs and can lead to aggressive encounters with territory owners (Dale and Slagsvold 1995).

Several hypotheses about the function of extra-territorial foraying have been proposed. First, extra-territorial visits may function to search for mating opportunities (Double and Cockburn 2000; Westneat and Stewart 2003). In reed buntings, *Emberiza schoeniclus*, and blue tits, *Cyanistes caeruleus*, for instance, males that had made visits to a female’s territory were more likely to sire extrapair offspring with her (Kleven et al. 2006; Schlicht et al. 2015; but see Celis-Murillo et al. 2017). In species in which reproduction is monopolized by a dominant individual, such as meerkats *Suricata suricatta*, such movements may even be the primary route to breeding for subordinates (Young et al. 2007; Mares et al. 2014). Second, extra-territorial visits may function to gather information about the suitability of potential future nest sites, either through direct information (e.g. about habitat or nest site quality) or through social information (e.g. reproductive success of local breeders) (Boulinier and Danchin 1997). Evidence for this hypothesis comes from studies on collared flycatchers *Ficedula albicollis* and blue tits, showing that individuals were more likely to disperse to areas where reproductive output had been experimentally enhanced (Doligez et al. 2002; Parejo et al. 2007). Other proposed functions of extra-territorial movements are searching for food (Westneat 1993; Neudorf et al. 1997; Kamler et al. 2019), assessing opportunities for intraspecific brood parasitism (Rohwer and Freeman 1989), and obtaining social information to inform reproductive investment decisions (Brandl et al. 2018, 2019).

Extra-territorial movements, and especially visits to nests of conspecifics, are not often observed. Male red-winged blackbirds, *Agelaius phoeniceus*, for instance, spent less than 6% of their active time away from their territory (Westneat 1993), and in blue tits, only 8% of females and 20% of males made at least one visit to a nest-box outside their territory during the fertile period (Schlicht et al. 2015). Moreover, individuals engaging in extra-territorial forays typically behave unobtrusively and remain silent, making them difficult to detect (Hanski and Haila 1988; Westneat 1988; Stutchbury 1998; Naguib et al. 2001; Woolfenden et al. 2005). As a consequence, studies on extra-territorial behavior are usually based on small sample sizes (Schlicht et al. 2015) and relatively few studies have investigated spatial and temporal patterns of this behavior at the population level (del Mar Delgado et al. 2011; Ponchon et al. 2013).

Recent technological developments have greatly improved opportunities for automated collection of behavioral data (Smith and Pinter-Wollman 2021). This now enables researchers to obtain data on infrequent or elusive behaviors while at the same time reducing the potential for interference by human observers, and has resulted in a number of studies that investigated foraying behavior on a larger scale. A study on western bluebirds, *Sialia mexicana*, for instance, investigated movements between territories using a radiofrequency identification (RFID) based system over three consecutive breeding seasons (Smith et al. 2020). A study on great tits, *Parus major*, also used RFID technology to track nest-box visits of 80 individuals during the pre-breeding and early breeding period (Firth et al. 2018). Another study on great tits investigated individual movements with a radio-tracking system of 66 birds throughout one breeding season (Bircher et al. 2020). Yet, population-wide data on extra-territorial visits throughout the breeding period are still scarce and the function of this behavior remains poorly understood.

Here, we investigated spatiotemporal patterns of extra-territorial visits to nest-boxes in a wild blue tit population. Blue tits are small, cavity-nesting passerine birds that breed throughout much of Europe (Gosler et al. 2020). During the breeding season, males and females defend a territory and aggressively chase away same-sex individuals from the vicinity of the nest-box (Kempenaers 1994). Nevertheless, nest-boxes already occupied by a breeding pair are occasionally visited by blue tits from other territories (Schlicht et al. 2015). We used an RFID-based monitoring system to record all nest-box visits over six breeding seasons (*n* = 1195 individual breeding attempts) and recorded a total of 22 137 extra-territorial visits. Here, our objective is to provide a comprehensive account of spatial and temporal patterns of extra-territorial nest-box visits in blue tits and examine the influence of individual-specific traits (sex and age class). We also test whether the occurrence of nest-box visits by individuals known to have an active nest predicts patterns of extrapair paternity and breeding dispersal. We then evaluate how these patterns align with previously proposed hypotheses for the function of extra-territorial nest visits.

## MATERIALS AND METHODS

### Study system

Data were collected between 2015 and 2020 in the Westerholz forest in Southern Germany (48° 08ʹ 26″ N 10° 53ʹ 29″ E), where we have studied the breeding behavior of blue tits since 2007. The forest is dominated by mature oak trees, and contains 277 nest-boxes of which between 112 and 195 were occupied each year (2015–2020). Boxes were distributed in a grid-like fashion, with an average distance between a box and its neighboring (i.e. four closest) boxes of 39 m ± 7 SD. In each spring, we visited all boxes at least once a week (where necessary daily) to monitor the onset and progress of nest building, including the date of the first egg, clutch size, the date of first hatching, and breeding success. For a more detailed description of the study site and general field procedures, see Schlicht et al. (2012).

Blue tits are socially monogamous, but extrapair paternity is common, with about half of the broods in our population containing at least one extrapair young (Schlicht et al. 2014; Santema et al. 2019, 2020). Extrapair copulations in blue tits are thought to cease around the onset of egg laying, as the majority of extrapair sired eggs are laid early in the laying sequence (Magrath et al. 2009; Valcu et al. 2019; Santema et al. 2021). Only females incubate the eggs, but both parents contribute extensively to offspring provisioning throughout the nestling phase (Perrins 1979). Blue tits at our study site breed only once a year, although some individuals produce a replacement clutch after their first breeding attempt failed at an early stage.

### Nest-box visit monitoring system

Nest-boxes were equipped with an automated monitoring system, consisting of a radio frequency identification (RFID) reader, a clock, and a data storage device (Loes et al. 2019). This system is a permanent feature of all nest-boxes at our study site (*n* = 277) and functions year-round. Each bird in the study was equipped with a passive integrated transponder (SMARTRAC glass tag 134 kHz, EM4305, 8.3 × 1.41 mm, 0.03 g) which was inserted on the back under the skin (Schlicht and Kempenaers 2018). Whenever a tagged bird was present at the nest hole, its identity, and the associated time and date were automatically stored (see Schlicht et al. 2012 for details). Birds had been caught either during previous breeding attempts when provisioning offspring, or prior to the breeding season using mist-nets and snap-traps, or when roosting in a nest-box. We attempted to catch any untagged individual throughout the winter, such that 90% of all breeding individuals had been tagged by the onset of the breeding period. This provided uniquely comprehensive data on extra-territorial nest-box visits during the breeding season.

### Data processing

**Figure 1:**
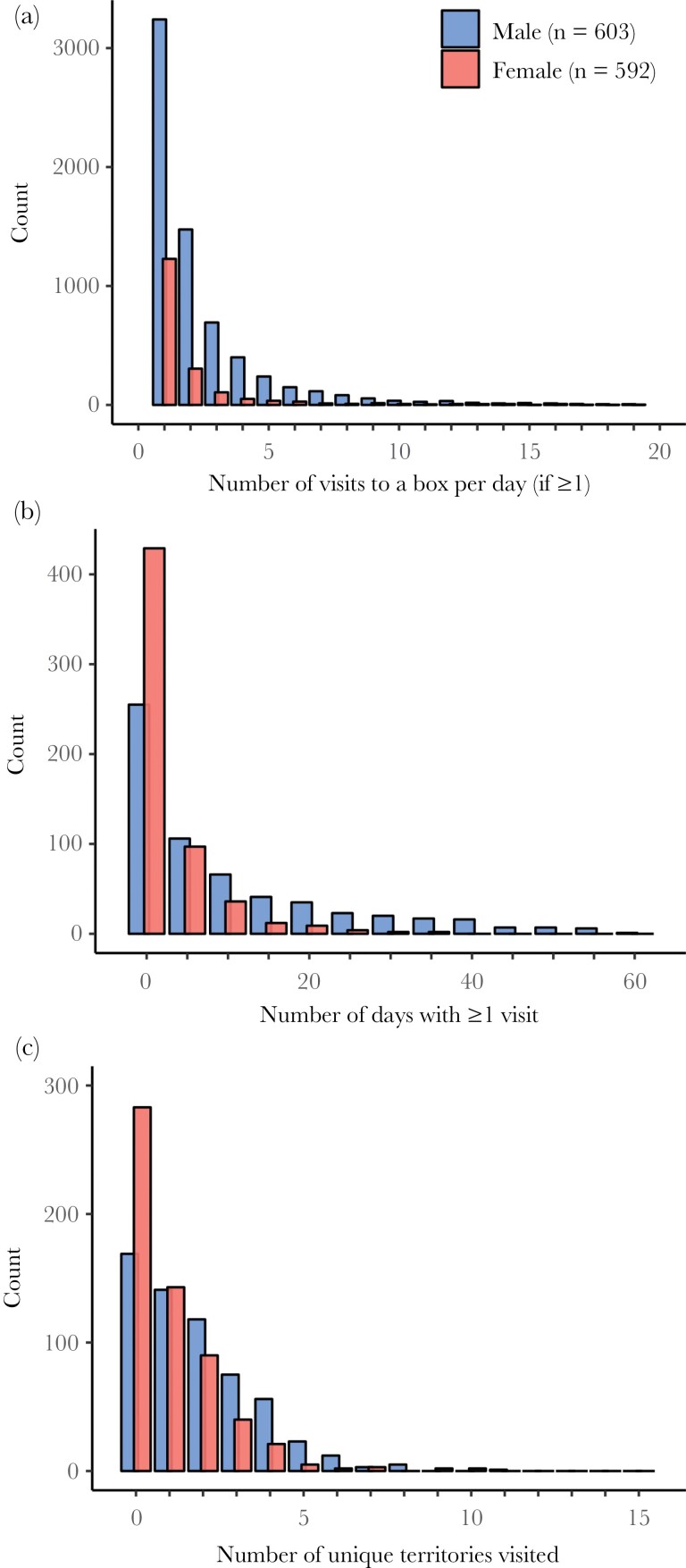
Distribution of (a) the number of visits of an individual male or female blue tit to a particular nest-box per day (only individuals that made at least one visit are included), (b) the number of days on which an individual made at least one extra-territorial nest-box visit, and (c) the number of different territories visited by an individual during the breeding season.

We extracted all visits made by a breeding individual to a nest-box in the territory of another pair. A “visit” refers to a reading by the RFID system, which implies that the tagged bird entered the nest-box or landed at the entrance hole. Readings close together in time cannot be considered independent visits and we therefore considered readings within a 10-minute window to be a single visit. Territory boundaries were determined using Thiessen polygons (Valcu and Kempenaers 2010). Because only a subset of the available nest-boxes was occupied every year, territories contained on average 0.9 (range = 0–4) unoccupied boxes besides the breeding box. We only included visits by individuals that were known to have an active nest at the time of their visit, to ensure that we did not include any pre- or post-breeding movements. That is, we only included visits made by an individual after they started nest building and before their offspring had fledged. We excluded visits made by individuals after their breeding attempt failed, as territoriality may break down after nest failure and subsequent visits are thus not necessarily true extra-territorial visits. We also excluded visits made by failed breeders before their nest failed, because 1) it is often not possible to determine when a nest failed and 2) by excluding failed breeders we ensured that all breeding stages are represented equally in our data. In the majority of cases where an individual visited another box on a particular day, it visited only once (50% of cases) and an individual rarely visited a box more than ten times in a day (<4% of cases, Figure 1a). For most analyses we therefore only assessed whether an individual had made at least one visit on a particular day. Birds that were transponder-implanted after the onset of the breeding attempt (10% of total number of breeders) were excluded from analyses.

### Paternity analysis

Blood samples (ca. 5–10 µl) were taken from all adults when they were caught for the first time and from all nestlings when they were banded and measured at fourteen days of age. We also collected dead nestlings and unhatched eggs and genotyped them if sufficient DNA could be extracted. In total, we genotyped 1013 breeders (456 males, 557 females) and 7320 offspring from 856 nests over the study period. To assess parentage, we compared the genotypes from parents and their putative offspring using a set of 14 microsatellite markers following the procedures described in Schlicht et al. (forthcoming).

### Statistical analyses

All data processing was performed with the software R (version 4.1.0; R Development Core Team 2021). All statistical modeling was performed using the lme4 package (Bates et al. 2015) and figures were prepared using the ggplot2 package (Wickham 2016).

To examine factors affecting the likelihood of making at least one extra-territorial box visit on a particular day, we constructed a generalized linear mixed model (GLMM) with a binomial error structure, with whether or not an individual made at least one visit to another box (yes/no) as the response variable. Sex (male or female), age (yearling or adult), and date (relative to onset of egg laying) were included as fixed effects. In addition, we included an interaction effect between sex and date, to test for differences in seasonal patterns between the sexes. We also included an interaction effect between age and sex, to test whether any effect of age differs between the sexes. Finally, we included the 1st and 2nd-order polynomial of date to allow for a non-linear seasonal effect. Individual identity and year were included as random intercepts. For each individual (*n* = 1195), we included all days between the start of nest building and fledging, totaling 64 345 individual days. We also ran the same model with date relative to hatching instead of relative to start of egg laying, which yielded qualitatively the same results (Table S1).

To examine seasonal patterns in the likelihood of *receiving* at least one extra-territorial visit on a particular day, we ran a GLMM with a binomial error structure, and with whether or not a breeding pair received at least one visit (yes/no) as the response variable (Table S2). We included date (relative to the onset of egg laying or relative to hatching) as the fixed effect, and pair identity and year as random intercepts. We included the 1st and 2nd-order polynomial of date to allow for a non-linear seasonal effect. We ran the models separately for visits received from males or from females.

To examine factors influencing the likelihood of visiting a particular territory, we created a list of all combinations of individuals and territories and added whether the individual had visited that territory at least once during the breeding period (yes/no). Because blue tits rarely visited boxes that were separated by more than three territories (see Results), we excluded boxes that were separated by more than three territories. Specifically, we examined the influence of neighborhood order (i.e. whether the box belonged to a 1st order, 2nd order, or 3rd order neighbor) and distance to the breeding box (in meters). We constructed GLMMs (separately for males and females) with a binomial error structure, with whether an individual had made a visit (yes/no) as the response variable and with neighborhood order and distance as fixed effects. We included the 1st and 2nd-order polynomial of neighborhood order and distance to allow for a non-linear effect. Individual identity and year were included as random intercepts. Neighborhood order and distance were correlated (*r*_Pearson_ = 0.78). Because we were interested in assessing whether distance had an effect after accounting for neighborhood order (and *vice versa*), we estimated their effects simultaneously. Effect sizes were similar in models where only one of the variables was included (details not shown).

To test whether the occurrence or frequency of extra-territorial box visits of individuals was related to their likelihood of producing extrapair offspring, we performed GLMMs (for males and female separately) with whether an individual had sired at least one extrapair offspring (yes/no) as the response variable and whether or not an individual had performed at least one extra-territorial box visit as fixed effect. As age class is a strong predictor of extrapair siring success in males (Cleasby and Nakagawa 2012), we additionally included age (yearling or adult) as a fixed effect. Individual identity and year were included as random intercepts. We also ran a model with the number of days on which an individual had made at least one extra-territorial visit – instead of whether it had made at least one visit – as an explanatory variable and this yielded qualitatively the same results (Table S3).

For individuals that did have at least one extrapair offspring, we also tested whether the likelihood of having extrapair offspring with a particular opposite sex individual was predicted by whether they had visited the nest-box of that individual. To this end, we created a list of all combinations of males and females and added whether they had produced extrapair offspring together (yes/no). Because blue tits rarely have extrapair offspring with individuals that are separated by more than two territories (Schlicht et al. 2015), we excluded male–female combinations that were separated by more than two territories. We constructed GLMMs (separately for males and females) with a binomial error structure, with whether an individual had produced offspring with a particular opposite sex individual (yes/no) as the response variable and neighborhood order (i.e. whether the box belonged to a 1st or 2nd order neighbor) and whether the focal individual had visited the box of the opposite sex individual as fixed effects. Individual identity and year were included as random intercepts. We also ran a model with the number of days on which an individual had made at least one extra-territorial visit as an explanatory variable and this yielded qualitatively the same results (Table S4).

To test whether the occurrence or frequency of extra-territorial box visits of individuals was related to breeding dispersal, we extracted all cases where an individual bred in the study population in two consecutive years (*n* = 402). We determined for each individual whether it had performed at least one extra-territorial box visit (in year x) and whether or not it had dispersed (in year x + 1). We considered an individual to have dispersed if it had moved to a box that was more than 70 m from its previous box and that box was outside the male’s year × territory (estimated using Thiessen polygons). We performed GLMMs (for males and females separately) with whether or not an individual had dispersed as the response variable and whether it had made at least one extra-territorial visit as fixed effect. Individual identity and year were included as random intercepts. We also ran a model with the number of days on which an individual had made at least one extra-territorial visit as an explanatory variable and this yielded qualitatively the same results (Table S5).

For individuals that did disperse, we also tested whether the box to which they dispersed was predicted by whether or not they had visited the territory to which that box belonged. To this end, we created a list of all combinations of dispersed individuals and territories and added whether the individual had dispersed to a box in that territory (yes/no). Because blue tits rarely visit boxes separated by more than three territories or disperse over such distances (Valcu and Kempenaers 2008), we only considered boxes that were separated by no more than three territories. We constructed GLMMs (separately for males and females) with a binomial error structure, with whether an individual had dispersed to a particular territory (yes/no) as the response variable and whether the focal individual had visited that territory as fixed effect. Neighborhood order (i.e. whether the box belonged to a 1st, 2nd, or 3rd order neighbor) was also included as a fixed effect. Individual identity and year were included as random intercepts. Additionally, we ran a model with the number of days on which an individual had made at least one extra-territorial visit as an explanatory variable and this yielded qualitatively the same results (Table S6).

By using Thiessen polygons to estimate territory boundaries, unoccupied boxes were assigned to the territory of the nearest occupied box. However, we have no information about the exact location of territory borders, and it is likely that real territories do not always conform to this rule. Some visits to empty boxes that we classified as extra-territorial may thus have been within an individual’s own territory, despite the fact that it was closer to the breeding box of another pair. We therefore additionally ran more conservative analyses of extra-territorial nest-box visit behavior that only included visits to the breeding box of another pair (*n* = 7834 visits, 35% of total). The results of these analyses are presented in the supplementary materials (Table S7-S12, Figure S1-S5). These more conservative analyses yielded mostly the same results and did not affect the main conclusions. Any qualitative differences between the analyses using all visits and those using visits to occupied boxes only are highlighted in the results section.

## RESULTS

### Description of extra-territorial visits

During the 1195 individual breeding attempts in this study (by 761 unique individuals) we recorded 22 137 extra-territorial nest-box visits. Sixty-two percent of individuals made at least one extra-territorial visit during their breeding attempt and the median number of days on which those individuals visited another nest-box was six (IQR: 2–14, Figure 1b). Of the 743 cases where an individual visited at least one other territory during its breeding attempt, 284 (38%) visited only one territory, 208 (28%) visited two, 115 (15%) visited three, 77 (10%) visited four and 59 (8%) visited five or more different territories (Figure 1c).

### Factors affecting whether individuals make visits

**Figure 2:**
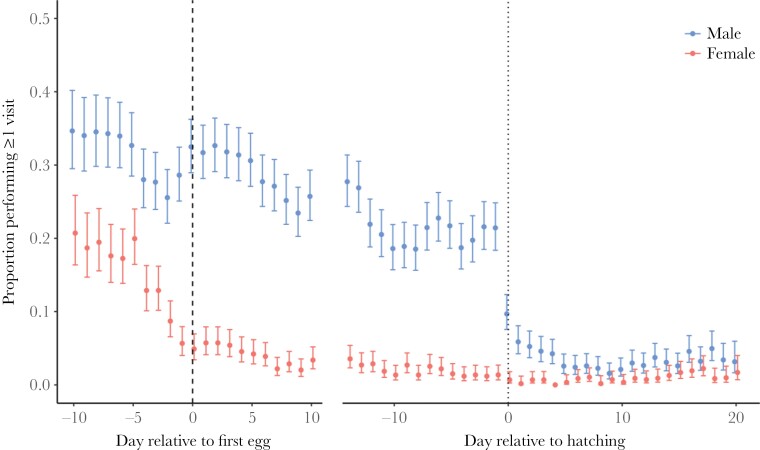
Daily probability of making at least one extra-territorial nest-box visit over the course of the breeding period for female (red) and male (blue) blue tits. Days early in the season are shown relative to the start of egg laying (until 10 days after the start of laying; the mean day of clutch completion). Days later in the season are shown relative to the start of hatching (starting 14 days before hatching; the mean day of incubation start). Dashed lines indicate the onset of laying and dotted lines indicate the start of hatching. Estimates for each day are derived from GLMMs in which date (relative to first egg or hatching) was included as a factor, such that the probability of a visit was estimated for each day separately. Points and error bars represent predicted values and 95% CIs.

There was a clear difference between the sexes in the likelihood of making visits, with males making more extra-territorial visits than females (Table 1, Figure 2). There was also a strong seasonal decline in the frequency of such visits, with both males and females reducing the likelihood of making a visit over the course of the season (Table 1, Figure 2). However, there was a significant interaction between date (relative to the start of laying) and sex, with females showing an earlier decline compared with males (Table 1, Figure 2). Females showed a strong reduction in their extra-territorial visits a few days before the onset of egg laying, whereas males continued making visits until much later in the season and showed a strong and sudden drop in visits on the day their offspring hatched. The analyses including only visits to occupied boxes showed that adults were more likely to make visits than yearlings (Table S6), but this was not the case when all extra-territorial box visits were considered (Table 1). When only visits to occupied boxes were considered, the difference between males and females in the likelihood of making visits was smaller (Figure 2 cf. Figure S2), indicating that females visit occupied boxes proportionally more.

**Table 1:** Summary of a GLMM examining the relation between sex, age (yearling or adult), and date (relative to day of first egg) on the likelihood that blue tits made at least one extra-territorial nest-box visit on a particular day. Analyses included data from all individuals (*n* = 1195). Explanatory variables with *P* < 0.05 are indicated in bold. See Methods for details

	Estimate	SE	*z*	*P*
*(Intercept)*	−1.96	0.15		
**Sex**	**−1.92**	**0.23**	**−8.39**	**<0.001**
Age	0.05	0.20	0.26	0.80
**Date (relative to egg laying)**	**−0.08**	**0.00**	**−52.97**	**<0.001**
**Date^2**	**−83.11**	**5.39**	**−15.42**	**<0.001**
**Sex × date**	**−0.02**	**0.00**	**−6.26**	**<0.001**
Sex × age	−0.21	0.31	−0.68	0.50

Age effect is relative to yearling, and sex effect is relative to male.

### Factors affecting whether a breeding pair received a visit

There was a strong seasonal pattern in the likelihood that a breeding pair received at least one visit by another territory holder (Table S2, Figure S6). The likelihood of receiving a visit from a female was highest in the period before egg-laying and declined gradually thereafter (Figure S6). The likelihood of receiving a visit from a male was the highest during the early egg-laying phase, with a peak on the third day after egg-laying and a gradual decline thereafter (Figure S6).

### Factors affecting visits to a particular territory

**Figure 3:**
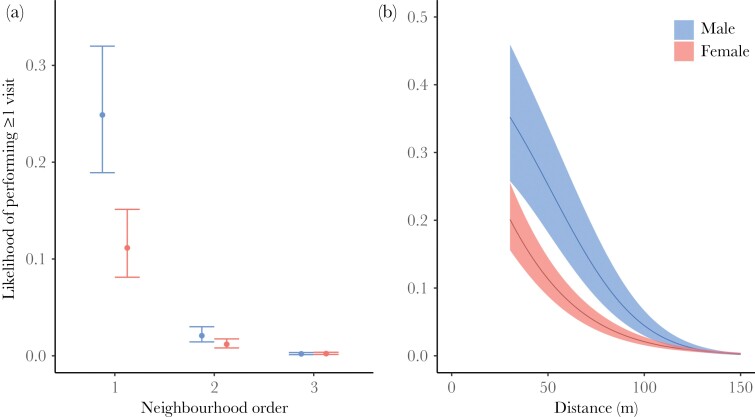
Relationship between whether or not a male (blue) or female (red) blue tit visited a particular territory at least once during the breeding period and (a) neighborhood order and (b) the distance to the breeding box. Estimates for each neighborhood order are derived from GLMMs in which neighborhood order was included as a factor, such that the probability of a visit was estimated for each level separately. Points and error bars represent predicted values and 95% CI.

Whether blue tits visited a particular territory at least once during the breeding period was strongly affected by the neighborhood order and the distance from the breeding box (Table 2, Figure 3). Males and females visited boxes of direct (1st order) neighbors with a likelihood of 28% and 15%, respectively, whereas the likelihood of visiting the nest-box of 3rd order neighbors was <1% (Figure 3a). Both males and females also rarely visited boxes more than 100 m from their breeding box (Figure 3b).

**Table 2:** Summary of a GLMM examining factors affecting whether or not males and females visited a particular box in another breeding pair’s territory at least once during the breeding period. Analyses included data from all individuals (*n*_males_ = 603, *n*_females_ = 592). Explanatory variables with *P* < 0.05 are indicated in bold. See Methods for details

		Estimate	SE	*z*	*P*
Males	*(Intercept)*	−5.26	0.26		
	**Neighborhood order**	**−126.18**	**15.23**	**−8.29**	**<0.001**
	**(Neighborhood order)** ^**2**^	**34.61**	**8.40**	**4.12**	**<0.001**
	**Distance**	**−295.73**	**34.90**	**−8.47**	**<0.001**
	**Distance** ^**2**^	**−103.68**	**17.71**	**−5.86**	**<0.001**
Females	*(Intercept)*	−5.08	0.20		
	**Neighborhood order**	**−97.45**	**15.08**	**−6.46**	**<0.001**
	**(Neighborhood order)** ^**2**^	**28.81**	**8.08**	**3.57**	**<0.001**
	**Distance**	**−164.87**	**30.54**	**−5.40**	**<0.001**
	Distance^2^	−29.65	16.90	−1.76	0.079

### Daily variation in visiting behavior

**Figure 4:**
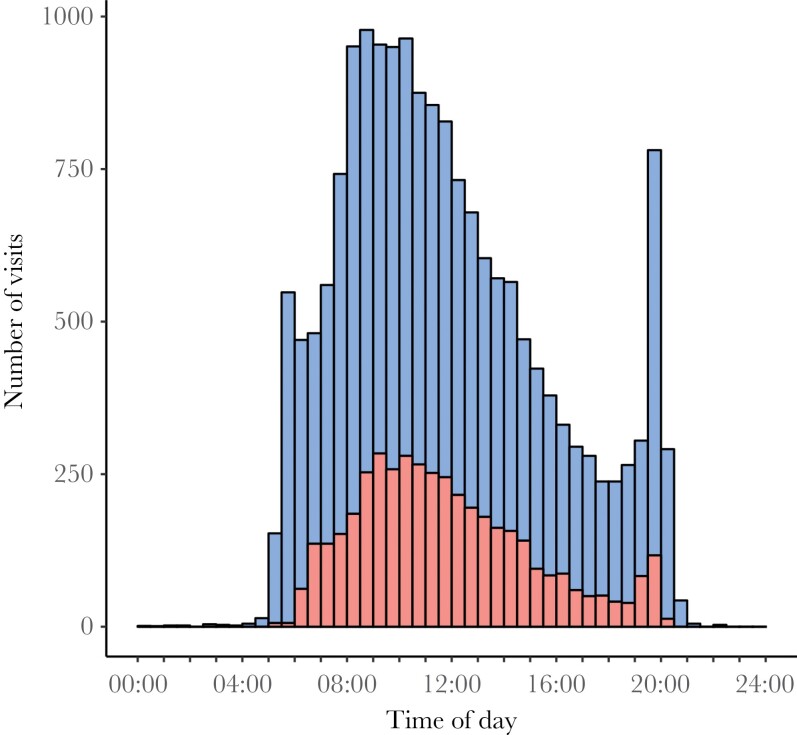
Frequency distribution of the time of day of visits by male (blue bars) and female (red bars) blue tits to boxes that were in another breeding pair’s territory.

Box visits were unevenly distributed across the day, with most visits taking place mid-morning (Figure 4). Both males and females also showed a pronounced peak in visit behavior in the evening (Figure 4). Males started visiting an extra-territorial nest-box earlier in the day and continued visiting later in the day compared with females (Figure 4). The majority of visits (92.4%) took place between sunrise and sunset, but a small proportion occurred shortly before sunrise (6.0%) or after sunset (1.6%).

### Consequences of extra-territorial box visits

**Figure 5:**
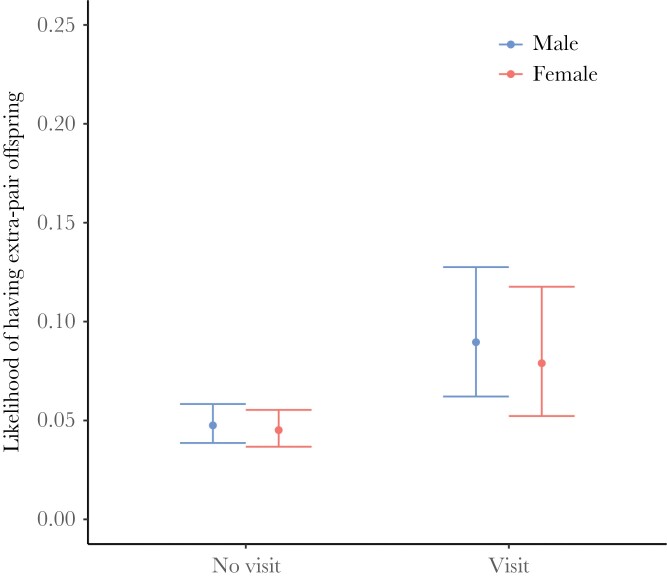
Relationship between whether or not a blue tit visited a particular box at least once during the breeding period and whether it produced extrapair young with the opposite sex individual of that box. We included all cases in which a successful breeder had produced at least one extrapair offspring with an opposite sex individual that was also included in the box visit dataset (*n*_males_ = 153, *n*_females_ = 153). Points and error bars represent predicted values and 95% CI derived from the models described in Table 4 c,d.

Males that made extra-territorial box visits were not more likely to sire extrapair young than males that had made no visits (Table 3). However, the likelihood that a male sired extrapair young with a particular female was predicted by whether he had visited the territory of that female (after accounting for neighborhood order, Table 4, Figure 5). Similarly, females that made extra-territorial box visits were not more likely to have extrapair offspring in their brood than females that had made no extra-box visits (Table 3), but the likelihood of having extrapair young with a particular male was predicted by whether she had visited the territory of that male (Table 4, Figure 5). The effect for females was not significant when only visits to occupied boxes were considered, although the trend was in the same direction (Table S9, Figure S5). Consistent with other studies on blue tits and other songbirds (reviewed in Cleasby and Nakagawa 2012), adult males were more likely to sire extrapair young than yearling males (Table 3). Additionally, and consistent with previous work on blue tits (Schlicht et al. 2014), neighborhood order strongly predicted the likelihood of having extrapair young with a particular individual (Table 4).

**Table 3:** Summary of GLMMs examining whether the likelihood that a male or a female had produced extrapair offspring was associated with their age class (yearling or adult) and with whether they had performed at least one extra-territorial visit. Analyses included data from all individuals (*n*_males_ = 603, *n*_females_ = 592) Explanatory variables with *P* < 0.05 are indicated in bold

		Estimate	SE	*z*	*P*
Males	*(Intercept)*	−3.93	0.49		
	Visit	−0.02	0.22	−0.09	0.93
	**Age**	**1.76**	**0.24**	**7.20**	**<0.001**
Females	*(Intercept)*	−1.34	0.33		
	Visit	0.33	0.19	1.77	0.08
	Age	0.18	0.19	0.98	0.33

Age effect is relative to yearling.

**Table 4:** Summary of GLMMs examining factors associated with the likelihood that a male or a female had produced extrapair offspring with a particular opposite sex individual. Analyses included all cases in which a successful breeder had produced at least one extrapair offspring with an opposite sex individual that was also included in the box visit dataset (*n*_males_ = 153, *n*_females_ = 153). Explanatory variables with *P* < 0.05 are indicated in bold. See Methods for details

		Estimate	SE	*z*	*P*
Males	*(Intercept)*	−0.45	0.29		
	**Visited**	**0.68**	**0.20**	**3.37**	**0.001**
	**Neighborhood order**	**−1.55**	**0.20**	**−7.90**	**<0.001**
Females	*(Intercept)*	−0.30	0.27		
	**Visited**	**0.60**	**0.22**	**2.67**	**0.008**
	**Neighborhood order**	**−1.66**	**0.19**	**−8.82**	**<0.001**

There were 402 (200 males, 202 females) cases where an individual bred in two consecutive years. Of these, 44 (8 males, 36 females) had dispersed; that is they nested in a box that was outside their previous territory (estimated through Thiessen polygons) and more than 70 m from their previous box. Whether an individual had made at least one extra-territorial box visit did not predict the likelihood of dispersal for either males or females (Table 5). Also, for individuals that did disperse, the likelihood of moving to a particular territory was not predicted by whether or not they had visited that territory (Table 6).

**Table 5:** Summary of GLMMs examining whether the likelihood that a male or a female dispersed between breeding seasons was predicted by whether or not they had performed at least one extra-territorial box visit. Analyses include cases where an individual bred in two consecutive years (*n*_males_ = 200, *n*_females_ = 202)

		Estimate	SE	*z*	*P*
Males	*(Intercept)*	−27.94	238.08		
	Visited	24.94	238.08	0.11	0.92
Females	*(Intercept)*	−1.21	0.33		
	Visited	−0.56	0.38	−1.49	0.14

**Table 6:** Summary of GLMMs examining factors associated with the likelihood that a male or a female had dispersed to a particular territory. Analyses included individuals that dispersed to a box outside their previous territory and more than 70 m from their previous box (*n*_males_ = 8, *n*_females_ = 36), except those that dispersed >3 territories away (*n* = 5) because visits to such distances are rare. Explanatory variables with *P* < 0.05 are indicated in bold. See Methods for details

		Estimate	SE	*z*	*P*
Males	*(Intercept)*	−3.23	1.46		
	Visited	1.58	1.05	1.51	0.13
	Neighborhood order	−0.13	0.60	−0.21	0.83
Females	*(Intercept)*	−0.85	0.47		
	Visited	0.07	0.79	0.09	0.93
	**Neighborhood order**	**−1.31**	**0.27**	**−4.89**	**<0.001**

## DISCUSSION

We investigated extra-territorial nest-box visits in a blue tit population over six breeding seasons, including 1195 individual breeding attempts and a total of 22 137 visits. There were some clear patterns: males performed more visits outside their territory than females and there was strong seasonal effect, with both males and females performing most visits early in the breeding period. However, females reduced their visit behavior before the start of egg laying, whereas males continued until later and showed a strong drop when their eggs hatched. Almost all visits were to first order neighbors, and individuals rarely visited boxes further away. Below, we evaluate our findings in the light of previously proposed hypotheses about the function of extra-territorial movements.

Several aspects of our results support the hypothesis that extra-territorial visits serve a function in extrapair mating behavior (Double and Cockburn 2000; Westneat and Stewart 2003). Males made many more visits than females and continued making them for longer, which is consistent with the fact that males benefit more from extrapair copulations than females (Arnqvist and Kirkpatrick 2005; Forstmeier et al. 2014) and can do so as long as there are fertile females in the population. Previous work on great tits also reported marked sex difference in seasonal trends, with females reducing their visit behavior before the start of laying and males continuing to make visits until after the start of laying (Firth et al. 2018). The finding that most visits were to first order neighbors is also consistent with this interpretation, as extrapair copulations are known to occur almost exclusively between close neighbors (Schlicht et al. 2014). Finally, we found that the likelihood that a male and female produced extrapair offspring together was predicted by whether or not they had visited each other’s territories. This had previously been shown in our population for males (Schlicht et al. 2015), but not for females. The fact that females started decreasing their visit rates already a few days before the start of egg-laying (see also Ackay et al. 2012; Firth et al. 2018), that is the period when extrapair fertilizations typically take place (Magrath et al. 2009), suggests that male extra-territorial visits may be more important for extrapair fertilizations than female extra-territorial visits. Alternatively, females might reduce their investment in seeking extrapair copulations close to the start of laying. Further note that our study only records visits to nest-boxes, and these patterns may not hold for extra-territorial visits in general.

Breeding pairs were most likely to receive visits from males when the visited female had just started egg-laying. This pattern indicates that males are most likely to visit other territories during the fertile period of the local female. However, breeding pairs continued to receive visits from males – albeit at a lower and decreasing rate – throughout the egg-laying and incubation period. Such visits suggest either that males are not aware of the fertility status of the visited female, or that they perform extra-territorial nest-box visits for reasons other than seeking extrapair mating opportunities.

Our results are not consistent with the hypothesis that extra-territorial nest-box visits function to gather (social) information about potential future nest sites or dispersal opportunities. This hypothesis predicts that these visits should be most common towards the end of the breeding season when the relative quality of different territories can best be assessed (Boulinier et al. 1996), for instance based on the number or quality of offspring produced in other nests (Doligez et al. 2002, 2004). Blue tits showed the opposite pattern, with many nest-box visits early in the breeding season and few visits towards the end of the breeding season. Also, almost all visits were to nearby boxes, whereas prospecting behavior to inform dispersal decisions would not necessarily be expected to be within such a restricted range (Firth et al. 2018). Moreover, males performed many more visits than females, even though female blue tits are much more likely to disperse between breeding attempts than males (Valcu and Kempenaers 2008, this study). Finally, whether or not an individual performed extra-territorial visits did not predict whether it dispersed outside of its territory in the following year, and individuals that dispersed were not more likely to disperse to a territory that they had visited the previous year. Note that our study was restricted to nest visits by individuals with an active nest. Hence, we cannot exclude that prospecting for future nest sites plays a more important role for other individuals, such as failed breeders, non-breeders, and finished breeders. It seems unlikely that extra-territorial forays in blue tits are related to foraging outside the territory (Westneat 1993; Neudorf et al. 1997; Kamler et al. 2019). Food demands in blue tits are highest during the period of nestling provisioning, which is when they performed fewest extra-territorial visits. Also, food demands of females may be elevated during egg laying, but females reduced their foraying behavior already before they started egg laying. Note however, that we only recorded visits to a nest-box, so we cannot exclude that blue tits made forays into other territories for foraging purposes.

Regardless of its function, our results suggest that there may be a trade-off between extra-territorial behavior and parental behavior, as both sexes showed a reduction in extra-territorial visits when their parental responsibilities increased. Females showed a strong reduction in visit behavior just before they started egg laying and reduced their visit behavior further during the time of incubation and nestling provisioning. Males – who do not contribute to incubation – continued performing extra-territorial visits at a high rate throughout this period. However, they showed a strong and sudden drop in visit behavior on the day their eggs hatched, which is when they start provisioning at a high rate.

Although our data suggest a potential role for extra-territorial visits in (extra-pair) mating interactions, it remains unclear what birds do during extra-territorial nest-box visits. A previous study showed that males visit other nest-boxes disproportionally when the female is present in the nest-box (Schlicht et al. 2015), and visiting a female at her nest may increase a male’s chance of successfully acquiring a (forced) extrapair copulation (Boulton et al. 2018; Plaza et al. 2019). However, it is also possible that copulations take place elsewhere, and that visits to the nest simply reflect interactions with the neighboring female. Future work may benefit from complementing nest-box visit data with video recordings or behavioral observations to get a better understanding of this behavior. Also, more detailed examination of patterns of extra-territorial box visits during the period when most visits take place – that is, the early breeding period – would help further understand this behavior. For instance, do blue tits primarily visit other boxes when the opposite sex individual of that box is present? And do they primarily visit other boxes when the same sex individual of that box is absent? Also, whilst much work on (extra-pair) mating behavior has focused on the early morning (Poesel et al. 2006, 2011; Kempenaers et al. 2010; Santema et al. 2020), we note a pronounced peak of extra-territorial visits of both males and females in the evening. This suggests that the evening may be more important for extrapair mating activity than appreciated, which deserves further attention.

Previous studies have often focused on a potential role of extra-territorial visits in prospecting for future nest sites, and often found support for this hypothesis (e.g. Reed et al. 1999; Doligez et al. 2004). However, few studies have examined visit behavior during the fertile period or monitored extra-territorial movements throughout the breeding period. Those that have seem to support a role in seeking extrapair matings. For example, in western bluebirds, most visits were made by males and during the fertile period of females (Smith et al. 2020). However, other aspects of visit behavior in this species suggested a role in prospecting for nest sites, indicating that visiting behavior may serve more than one function, even within the same species (Smith et al. 2020). Also, a previous study on blue tits found that extra-territorial prospecting predicted extrapair success in males (Schlicht et al. 2015). In contrast, a recent study on great tits reported that extra-territorial visits did not occur primarily during the fertile period and did not predict the occurrence of extrapair paternity (Bircher et al. 2020). Yet, taken together, these recent studies suggest that searching for extrapair mating opportunities may be a common function of extra-territorial movements.

A limitation of our study is that only extra-territorial movements that involved a visit to a nest-box were recorded. It is likely that this only represents a small proportion of all extra-territorial visits, and the actual frequency of extra-territorial movements may thus be considerably higher than suggested by our data. A recent study on great tits – a close relative of the blue tit – recorded all extra-territorial visits to within 15 m from other nest-boxes and reported that individuals typically made several visits per day (Bircher et al. 2020); substantially more than what we found in our study. Moreover, it is possible that the proportion of extra-territorial movements that involves a visit to another nest-box differs between sex and age classes or changes over the season. Actual patterns of extra-territorial movements would then not be well represented by our data. Indeed, Bircher et al. (2020) report that visits to within 15 m of another nest-box by both male and female great tits were most frequent during the nestling period, which contrasts with the seasonal patterns of visits to nest-boxes that we report. Continuous monitoring of individuals throughout their range would be required for a more comprehensive picture of extra-territorial movements, but this is challenging, especially in small free-living animals.

In summary, automated monitoring of nest-box visits in a blue tit population over six breeding seasons revealed some notable spatial and temporal patterns of extra-territorial nest visits. Almost all visits took place during the early breeding period and very few visits took place during nestling provisioning. This general seasonal pattern differed between the sexes, however, with females reducing their visits much earlier in the season than males. Almost all visits were to boxes in the immediate neighborhood and blue tits rarely made visits to territories further away. Extra-territorial box visits predicted patterns of extrapair paternity, but not patterns of breeding dispersal. Our results are not consistent with the hypothesis that such visits serve to inform dispersal decisions, but suggest that visits may be linked to extrapair mating opportunities. More detailed behavioral observations during extra-territorial visits are needed to understand what birds do when they visit another nest.

## Supplementary Material

arac111_suppl_Supplementary_Material

## Notes

We are grateful to Agnes Türk, Andrea Wittenzellner and all other people who contributed to the field work for data collection, to Peter Loës and Peter Skripsky for development and maintenance of the smart nestbox system, to Mihai Valcu for managing the database, and to Ben Sheldon and Maren Vitousek for discussion. We thank the editor Diego Gill, and Ross Crates and an anonymous reviewer for constructive comments on an earlier version of the manuscript. This work was generously supported by the Max Planck Society (to B.K.).

Authors’ Contribution: B.K. designed the methodology to collect the data; P.S. and B.K. conceived the ideas and designed the study; P.S. analyzed the data and wrote the manuscript with input from B.K.

Data Availability: Analyses reported in this article can be reproduced using the data provided by Santema & Kempenaers (2022).
