# Supplementary material for: Patterns of extra-territorial nest-box visits in a songbird suggest a role in extrapair mating
Source: Behav Ecol. 2022 Dec 23;34(1):150–9. doi: 10.1093/beheco/arac111 (PMC9918859; doi:10.1093/beheco/arac111)
Supplement: arac111_suppl_Supplementary_Material [file arac111_suppl_supplementary_material.docx]

Figure S1. Distribution of a) the number of visits that an individual male or female made to a particular box on one day (only individuals that made at least one visit are included), b) the number of days on which an individual made at least one extra-territorial nestbox visit, and c) the number of different territories visited by an individual during the breeding season. Figure differs from Figure 1 in the main text in that only visits to occupied boxes are included.


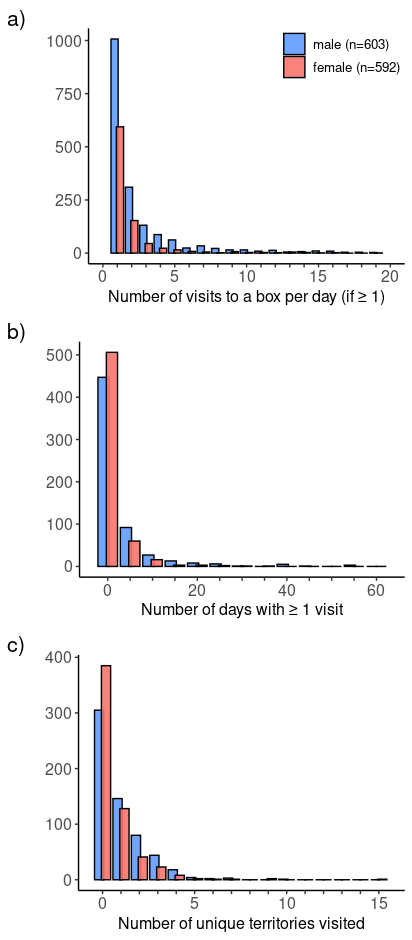


Figure S2. Daily probability of making at least one extra-territorial nestbox visit over the course of the breeding period for female (red) and male (blue) blue tits. Days early in the season are shown relative to the start of egg laying (until 10 days after the start of laying; the mean day of clutch completion). Days later in the season are shown relative to the start of hatching (starting 14 days before hatching; the mean day of incubation start). Dashed lines indicate the onset of laying and dotted lines indicate the start of hatching. Estimates for each day are derived from GLMMs in which date (relative to first egg or hatching) was included as a factor, such that the probability of a visit was estimated for each day separately. Points and error bars represent predicted values and 95% CI. Figure differs from Figure 2 in the main text in that only visits to occupied boxes are included.


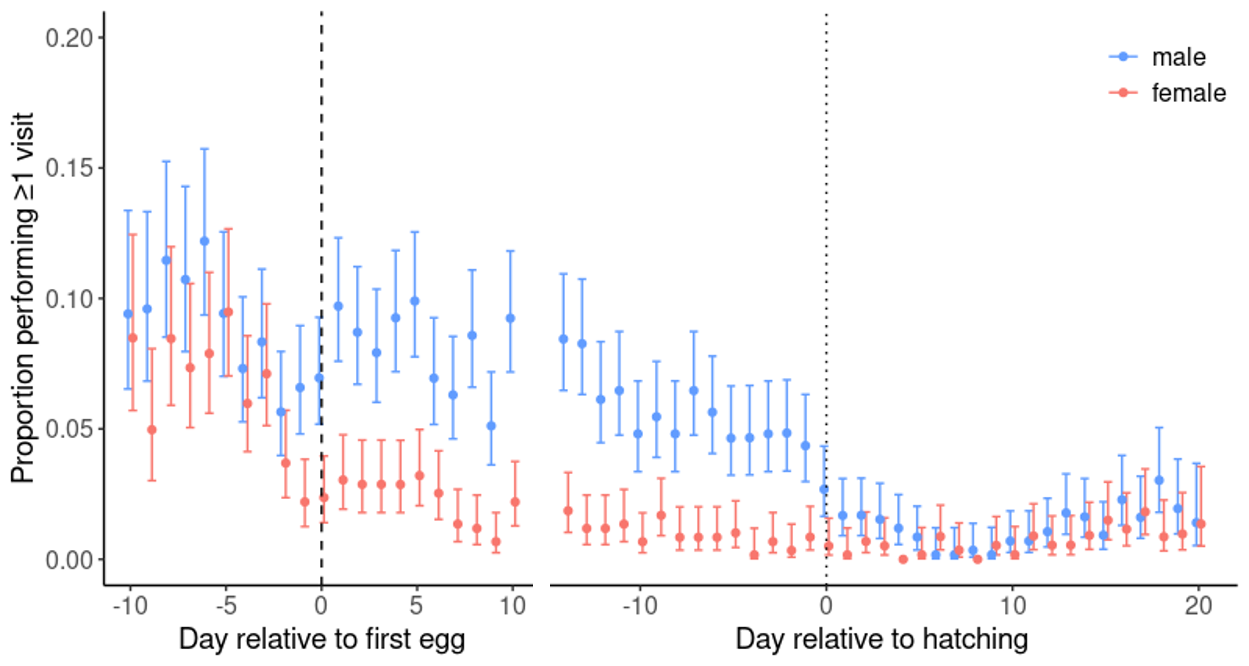


Figure S3. Relationship between whether or not a male (blue) or female (red) visited a particular territory at least once during the breeding period and a) neighborhood order and b) the distance to the breeding box. Estimates for each neighborhood order are derived from GLMMs in which neighborhood order was included as a factor, such that the probability of a visit was estimated for each level separately. Points and error bars represent predicted values and 95% CI. Figure differs from Figure 3 in the main text in that only visits to occupied boxes are included.


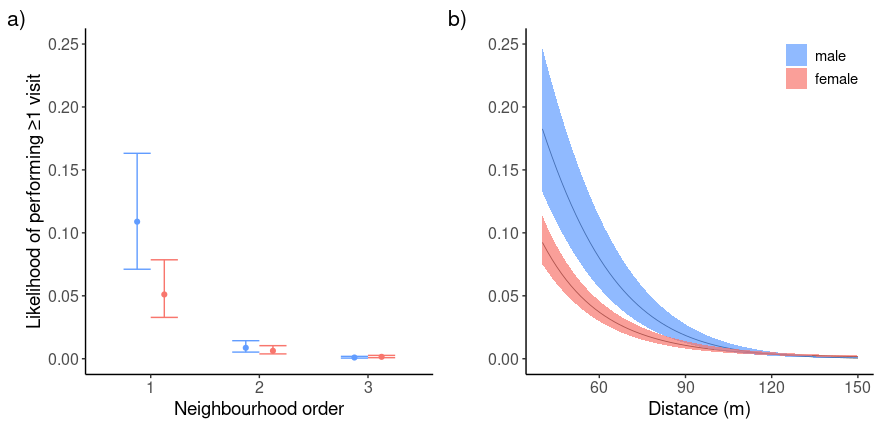


Figure S4. Frequency distribution of the time of day of visits by males (blue bars) and females (red bars) to boxes that were occupied by another breeding pair. Figure differs from Figure 4 in the main text in that only visits to occupied boxes are included.


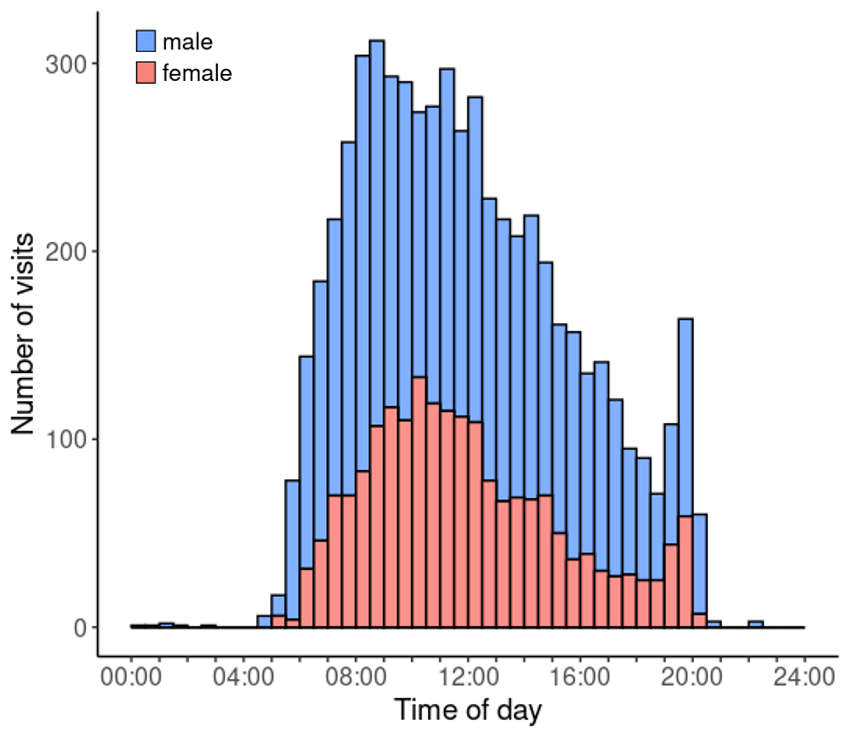


Figure S5. Relationship between whether or not an individual visited a particular box at least once during the breeding period and whether it produced extra-pair young with the opposite sex individual of that box. We included all cases in which a successful breeder had produced at least one extra-pair offspring with an opposite sex individual that was also included in the box visit dataset (n_males_=153, n_females_=153). Points and error bars represent predicted values and 95% CI derived from the models described in table 4 c,d. Figure differs from Figure 5 in the main text in that only visits to occupied boxes are included.


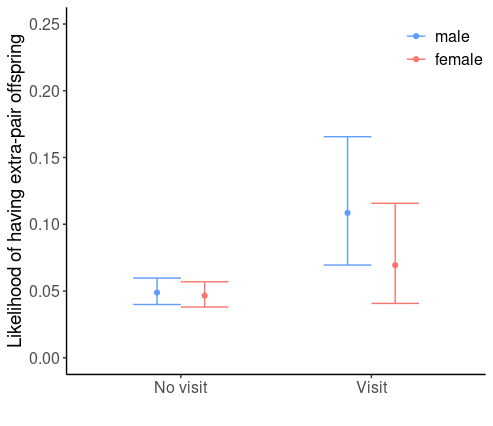


Figure S6. Daily probability that a breeding pair received at least one nestbox visit by another female (red) or male (blue) territory holder during the breeding period. Days early in the season are shown relative to the start of egg laying (until 10 days after the start of laying; the mean day of clutch completion). Days later in the season are shown relative to the start of hatching (starting 14 days before hatching; the mean day of incubation start). Dashed lines indicate the onset of laying and dotted lines indicate the start of hatching. Estimates for each day are derived from GLMMs in which date (relative to first egg or hatching) was included as a factor, such that the probability of a visit was estimated for each day separately. Points and error bars represent predicted values and 95% CIs.


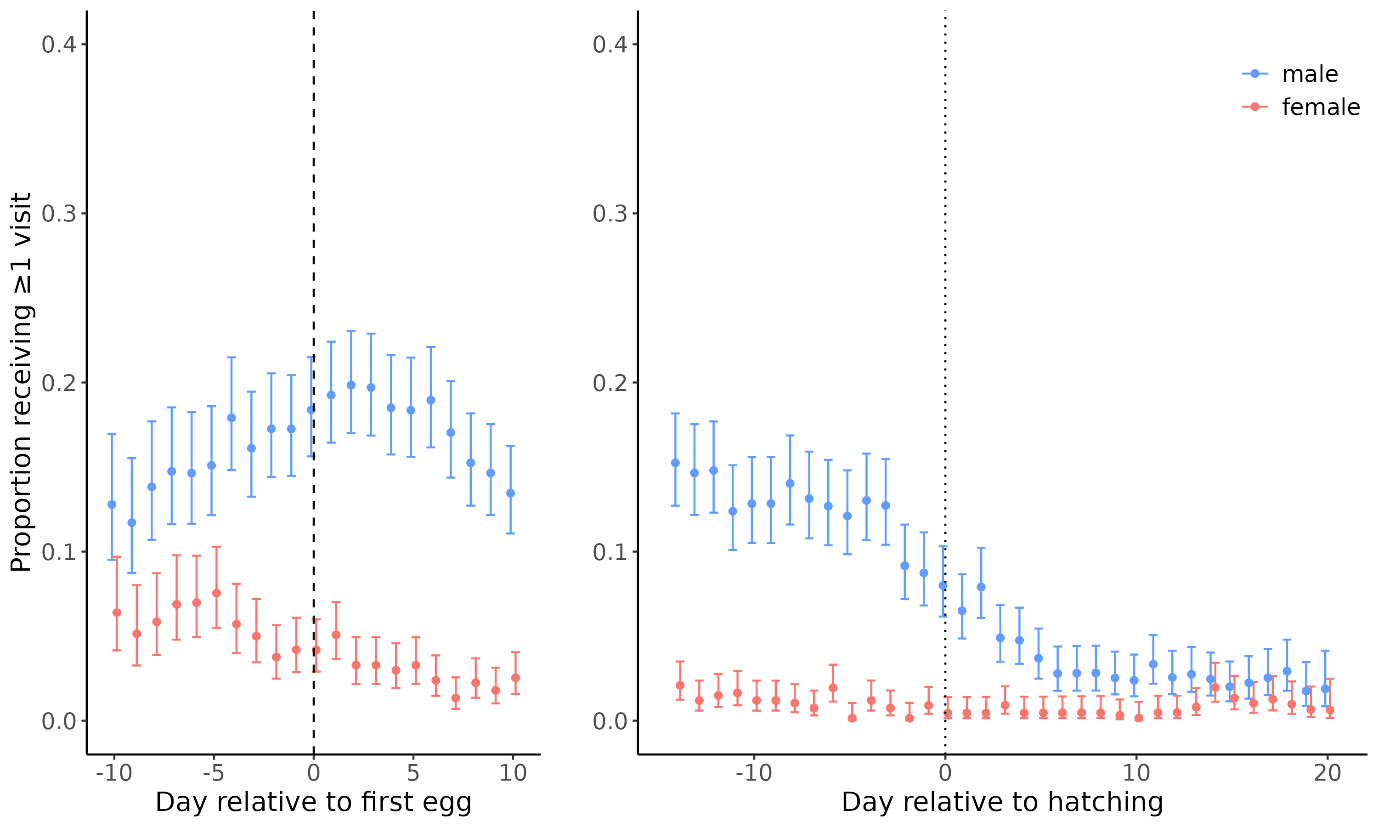


Table S1. Summary of a GLMM examining the relationship between sex, age, and date (relative to hatching) on the likelihood that blue tits made at least one extra-territorial nestbox visit on a particular day. Analyses included data from all individuals (n=1195). The model is the same as the one presented in table 1 in the main text, with the difference that date relative to hatching is used as an explanatory variable instead of date relative to the onset of egg-laying.

|  | estimate | se | z | p |
| --- | --- | --- | --- | --- |
| *(Intercept)* | -3.91 | 0.16 | -24.81 |  |
| **sex** | **-2.45** | **0.24** | **-10.18** | **<0.001** |
| age | 0.07 | 0.21 | 0.33 | 0.74 |
| **date (relative to hatching)** | **-0.08** | **0.00** | **-53.99** | **<0.001** |
| **date^2** | **-99.79** | **3.91** | **-25.51** | **<0.001** |
| **sex x date** | **-0.02** | **0.00** | **-7.36** | **<0.001** |
| sex x age | -0.24 | 0.31 | -0.78 | 0.44 |
|  |  |  |  |  |

Age effect is relative to yearling and sex effect is relative to male.

Table S2. Summary of GLMMs examining the relationship between date and the likelihood that a breeding pair received at least one visit from another territory holder on a particular day. We modeled the likelihood of receiving a visit by a male or by a female separately. Date was included in the models either relative to the day of the first egg of the visited nest, or relative to the day of hatching of the visited nest.

|  |  | estimate | se | z | p |
| --- | --- | --- | --- | --- | --- |
| male visits | *(Intercept)* | -3.89 | 0.11 | -36.54 |  |
|  | **date (relative to egg laying)** | **-189.05** | **5.18** | **-36.52** | **<0.001** |
|  | **date^2** | **-121.09** | **6.31** | **-19.20** | **<0.001** |
|  |  |  |  |  |  |
|  | *(Intercept)* | -3.94 | 0.11 | -37.04 |  |
|  | **date (relative to hatching)** | **-196.33** | **4.37** | **-44.98** | **<0.001** |
|  | **date^2** | **-137.84** | **4.27** | **-32.26** | **<0.001** |
|  |  |  |  |  |  |
| female visits | *(Intercept)* | -5.19 | 0.11 | -45.94 |  |
|  | **date (relative to egg laying)** | **-183.41** | **8.37** | **-21.92** | **<0.001** |
|  | **date^2** | **-10.16** | **6.99** | **-1.45** | **0.14** |
|  |  |  |  |  |  |
|  | *(Intercept)* | -5.18 | 0.13 | -41.03 |  |
|  | **date (relative to hatching)** | **-186.66** | **17.94** | **-10.41** | **<0.001** |
|  | **date^2** | **-12.37** | **11.49** | **-1.08** | **0.28** |
|  |  |  |  |  |  |

Table S3. Summary of GLMMs examining whether the likelihood that a male or a female had produced extra-pair offspring was associated with their age and the number of days on which they had performed at least one extraterritorial visit. Analyses included data from all individuals (n=603 males, 592 females). Models are similar as the one presented in table 3 of the main text, with the difference that the number of days on which an individual had made at least one extra-territorial visit – rather than whether or not an individual had made at least one visit – was included as explanatory variable.

|  |  | estimate | se | z | p |
| --- | --- | --- | --- | --- | --- |
| males | *(Intercept)* | -3.99 | 0.47 |  |  |
|  | visit days | 0.01 | 0.01 | 0.61 | 0.54 |
|  | **age** | **1.76** | **0.24** | **7.19** | **<0.001** |
|  |  |  |  |  |  |
| females | *(Intercept)* | -1.20 | 0.32 |  |  |
|  | visit days | 0.02 | 0.02 | 1.20 | 0.23 |
|  | age | 0.17 | 0.19 | 0.90 | 0.37 |
|  |  |  |  |  |  |

Age effect is relative to yearling.

Table S4. Summary of GLMMs examining factors associated with the likelihood that a male (c) or female (d) had produced extra-pair offspring with a particular opposite sex individual. Analyses included all cases where an individual that was included in the box visit dataset (i.e. a successful breeder) had produced ≥1 extra-pair offspring with an opposite sex individual that was also included in the box visit dataset (n=153 males, 153 females). Models are similar as the one presented in table 4 of the main text, with the difference that the number of days on which an individual had made at least one extra-territorial visit – rather than whether or not an individual had made at least one visit – was included as explanatory variable.

|  |  |  |  |  |  |
| --- | --- | --- | --- | --- | --- |
| males | *(Intercept)* | -0.19 | 0.26 |  |  |
|  | **visit days** | **0.11** | **0.03** | **3.40** | **0.001** |
|  | **neighborhood order** | **-1.67** | **0.19** | **-9.00** | **<0.001** |
|  |  |  |  |  |  |
| females | *(Intercept)* | -0.09 | 0.25 |  |  |
|  | visit days | 0.03 | 0.11 | 0.31 | 0.76 |
|  | **neighborhood order** | **-1.76** | **0.18** | **-9.56** | **<0.001** |
|  |  |  |  |  |  |

Table S5. Summary of GLMMs examining whether the likelihood that a male or female dispersed between breeding seasons was predicted by the number of days on which they had performed at least on extra-territorial box visit. Analyses include all cases where an individual bred in two consecutive years (n_males_=200, n_females_=202). Models are similar as the one presented in table 5 of the main text, with the difference that the number of days on which an individual had made at least one extra-territorial visit – rather than whether or not an individual had made at least one visit – was included as explanatory variable.

|  |  | estimate | se | z | p |
| --- | --- | --- | --- | --- | --- |
| males | *(Intercept)* | -8.72 | 1.35 |  |  |
|  | days visited | -0.07 | 0.07 | -0.98 | 0.33 |
|  |  |  |  |  |  |
| females | *(Intercept)* | -0.83 | 0.26 |  |  |
|  | days visited | -0.01 | 0.09 | -0.17 | 0.87 |
|  |  |  |  |  |  |

Table S6. Summary of GLMMs examining factors associated with the likelihood that a male or a female had dispersed to a particular territory. Analyses included individuals that dispersed to a box outside their previous territory and more than 70m from their previous box (n_males_=8, n_females_=36), except those that dispersed >3 territories away (n=5) because visits to such distances are very rare. See methods for details. Models are similar as the one presented in table 5 of the main text, with the difference that the number of days on which an individual had made at least one extra-territorial visit – rather than whether or not an individual had made at least one visit – was included as explanatory variable.

| males | *(Intercept)* | -2.36 | 1.06 |  |  |
| --- | --- | --- | --- | --- | --- |
|  | days visited | 0.11 | 0.07 | 1.43 | 0.15 |
|  | neighbourhood order | -0.44 | 0.49 | -0.90 | 0.37 |
|  |  |  |  |  |  |
| females | *(Intercept)* | -1.00 | 0.47 |  |  |
|  | days visited | 0.24 | 0.14 | 1.76 | 0.08 |
|  | **neighbourhood order** | **-1.25** | **0.27** | **-4.68** | **<0.001** |
|  |  |  |  |  |  |

Table S7. Summary of a GLMM examining the relation between sex, age, and date (relative to day of first egg) on the likelihood that blue tits made at least one extra-territorial nestbox visit on a particular day. Analyses included data from all individuals (n=1195). See methods for details. Analysis differs from the analysis presented in Table 1 of the main text in that only visits to occupied boxes are included.

|  | estimate | se | z | p |
| --- | --- | --- | --- | --- |
| *(Intercept)* | -3.75 | 0.16 |  |  |
| **sex** | **-0.96** | **0.23** | **-4.21** | **<0.001** |
| **age** | **-0.57** | **0.21** | **-2.72** | **0.006** |
| **date (relative to first egg)** | **-0.05** | **<0.00** | **-23.87** | **<0.001** |
| **date^2** | **-43.50** | **5.73** | **-7.59** | **<0.001** |
| **sex x date** | **-0.02** | **<0.00** | **-6.44** | **<0.001** |
| sex x age | 0.17 | 0.31 | 0.54 | 0.59 |
|  |  |  |  |  |

Age effect is relative to yearling and sex effect is relative to male.

Table S8. Summary of a GLMM examining factors affecting whether or not males and females visited a particular box occupied by another breeding pair at least once during the breeding period. Analyses included data from all individuals (n_males_=603, n_females_=592). See methods for details. Analyses differ from the analyses presented in Table 2 of the main text in that only visits to occupied boxes are included.

|  |  | estimate | se | z | p |
| --- | --- | --- | --- | --- | --- |
| males | *(Intercept)* | -6.29 | 0.27 |  |  |
|  | **neighborhood order** | **-51.96** | **15.23** | **-3.41** | **<0.001** |
|  | **neighborhood order^2** | **23.59** | **9.73** | **2.43** | **0.015** |
|  | **distance** | **-346.74** | **24.56** | **-14.12** | **<0.001** |
|  | **distance^2** | **-40.46** | **14.18** | **-2.85** | **<0.001** |
|  |  |  |  |  |  |
| females | *(Intercept)* | -5.54 | 0.21 |  |  |
|  | **neighborhood order** | **-39.96** | **14.33** | **-2.79** | **0.005** |
|  | neighborhood order^2 | 7.95 | 9.49 | 0.84 | 0.40 |
|  | **distance** | **-142.63** | **16.89** | **-8.44** | **<0.001** |
|  | **distance^2** | **54.40** | **10.85** | **5.02** | **<0.001** |
|  |  |  |  |  |  |

Table S9. Summary of GLMMs examining whether the likelihood that a male or a female had produced extra-pair offspring was associated with their age and with whether they had performed at least one extra-territorial visit. Analyses included data from all individuals (n_males_=603, n_females_=592). Analyses differ from the analyses presented in Table 3 of the main text in that only visits to occupied boxes are included.

|  |  | estimate | se | z | p |
| --- | --- | --- | --- | --- | --- |
| males | *(Intercept)* | -3.78 | 0.47 |  |  |
|  | visit | -0.27 | 0.21 | -1.32 | 0.19 |
|  | **age** | **1.73** | **0.25** | **7.05** | **<0.001** |
|  |  |  |  |  |  |
| females | *(Intercept)* | -1.29 | 0.33 |  |  |
|  | visit | 0.28 | 0.19 | 1.49 | 0.14 |
|  | age | 0.20 | 0.19 | 1.05 | 0.30 |
|  |  |  |  |  |  |

Age effect is relative to yearling.

Table S10. Summary of GLMMs examining factors associated with the likelihood that a male or a female had produced extra-pair offspring with a particular opposite sex individual. Analyses included all cases in which a successful breeder had produced at least one extra-pair offspring with an opposite sex individual that was also included in the box visit dataset (n_males_=153, n_females_=153). See methods for details. Analyses differ from the analyses presented in Table 4 of the main text in that only visits to occupied boxes are included.

| males | *(Intercept)* | -0.31 | 0.27 |  |  |
| --- | --- | --- | --- | --- | --- |
|  | **visited** | **0.86** | **0.25** | **3.50** | **<0.001** |
|  | **neighborhood order** | **-1.62** | **0.19** | **-8.55** | **<0.001** |
|  |  |  |  |  |  |
| females | *(Intercept)* | -0.16 | 0.26 |  |  |
|  | visited | 0.42 | 0.28 | 1.49 | 0.14 |
|  | **neighborhood order** | **-1.73** | **0.19** | **-9.31** | **<0.001** |
|  |  |  |  |  |  |

Table S11. Summary of GLMMs examining whether the likelihood that a male or a female dispersed between breeding seasons was predicted by whether or not they had performed at least on extra-territorial box visit. Analyses include cases where an individual bred in two consecutive years (n_males_=200, n_females_=202). Analyses differ from the analyses presented in Table 5 of the main text in that only visits to occupied boxes are included.

|  |  | estimate | se | z | p |
| --- | --- | --- | --- | --- | --- |
| males | *(Intercept)* | -3.84 | 0.75 |  |  |
|  | visited | 0.97 | 0.83 | 1.17 | 0.24 |
|  |  |  |  |  |  |
| females | *(Intercept)* | -7.72 | 1.28 |  |  |
|  | visited | -2.13 | 1.32 | -1.62 | 0.11 |
|  |  |  |  |  |  |

Table S12. Summary of GLMMs examining factors associated with the likelihood that a male or a female had dispersed to a particular territory. Analyses included individuals that dispersed to a box outside their previous territory and more than 70m from their previous box (n_males_=8, n_females_=36), except those that dispersed >3 territories away (n=5) because visits to such distances are very rare. Analyses differ from the analyses presented in Table 6 of the main text in that only visits to the occupied boxes are included.

|  |  | estimate | se | z | p |
| --- | --- | --- | --- | --- | --- |
| males | *(Intercept)* | -2.57 | 1.19 |  |  |
|  | visited | 1.28 | 1.02 | 1.25 | 0.21 |
|  | neighborhood order | -0.36 | 0.52 | -0.69 | 0.49 |
|  |  |  |  |  |  |
| females | *(Intercept)* | -0.77 | 0.46 |  |  |
|  | visited | -14.06 | 742.86 | -0.02 | 0.98 |
|  | **neighborhood order** | **-1.34** | **0.26** | **-5.07** | **<0.001** |
|  |  |  |  |  |  |
